# Genetic mapping of the powdery mildew resistance gene *Pm7* on oat chromosome 5D

**DOI:** 10.1007/s00122-023-04288-z

**Published:** 2023-03-13

**Authors:** Sophie Brodführer, Volker Mohler, Melanie Stadlmeier, Sylwia Okoń, Steffen Beuch, Martin Mascher, Nicholas A. Tinker, Wubishet A. Bekele, Bernd Hackauf, Matthias Heinrich Herrmann

**Affiliations:** 1 Federal Research Centre for Cultivated Plants, Institute for Breeding Research on Agricultural Crops, Julius Kuehn Institute (JKI), Rudolf-Schick-Platz 3a, OT Gross Lüsewitz, 18190 Sanitz, Germany; 2 Bavarian State Research Center for Agriculture, Institute for Crop Science and Plant Breeding, Am Gereuth 6, 85354 Freising, Germany; 3 Institute of Plant Genetics, Breeding and Biotechnology, University of Life Sciences in Lublin, Akademicka 15, 20-950 Lublin, Poland; 4 Nordsaat Saatzucht GmbH, Saatzucht Granskevitz, Granskevitz 3, 18569 Schaprode, Germany; 5 Research Group Domestication Genomics, Leibniz-Institut für Pflanzengenetik und Kulturpflanzenforschung (IPK), Corrensstraße 3, Stadt Seeland OT, 06466 Gatersleben, Germany; 6 Present Address: I.G. Saatzucht GmbH & Co KG, Am Park 3, 18276 Gülzow-Prüzen OT Boldebuck, Germany; 7 Agriculture and Agri-Food Canada, Ottawa Research and Development Centre, 960 Carling Ave., Ottawa, ON K1A 0C6 Canada

## Abstract

**Key message:**

Three independent experiments with different genetic backgrounds mapped the resistance gene *Pm7* in the oat genome to the distal part of the long arm of chromosome 5D.

**Abstract:**

Resistance of oat to *Blumeria graminis* DC. f. sp. *avenae* is an important breeding goal in Central and Western Europe. In this study, the position of the effective and widely used resistance gene *Pm7* in the oat genome was determined based on three independent experiments with different genetic backgrounds: genome-wide association mapping in a diverse set of inbred oat lines and binary phenotype mapping in two bi-parental populations. Powdery mildew resistance was assessed in the field as well as by detached leaf tests in the laboratory. Genotyping-by-sequencing was conducted to establish comprehensive genetic fingerprints for subsequent genetic mapping experiments. All three mapping approaches located the gene to the distal part of the long arm of chromosome 5D in the hexaploid oat genome sequences of OT3098 and ‘Sang.’ Markers from this region were homologous to a region of chromosome 2Ce of the C-genome species, *Avena eriantha*, the donor of *Pm7*, which appears to be the ancestral source of a translocated region on the hexaploid chromosome 5D.

**Supplementary Information:**

The online version contains supplementary material available at 10.1007/s00122-023-04288-z.

## Introduction

*Avena sativa* L. (*A*. *sativa*, oat) is appreciated for its high nutritional value in human diet and in livestock feeding. If oat is consumed on a regular basis, the high content of readily available β-glucan in the kernels can contribute to lower the risk of diet-related hypertension and cardiovascular diseases (Othman et al. 2011). While the absence of gluten makes oat unsuitable for baking bread, it offers a valuable alternative grain for people diagnosed with celiac disease (Ahola et al. 2020). Oat is also used as both a grain and green fodder for livestock production. In recent years, the growing area of oats in Europe has been low and fluctuating compared to 2008 (https://www.agrochart.com/en/usda/section/41/grains-eu/commodity/136/Oats-EU/attribute/1/area-harvested/), partly as a result of reduced use in livestock production. However, due to a steadily growing human consumption of oat products in Europe in the last two decades, there has been increased impetus for the cultivation and breeding of oats. Furthermore, the demand for uncontaminated foods and reductions in approved fungicides has made fungal resistance breeding an increasingly important tool in the development of new oat varieties. The introgression of resistance genes is important for pathogens that are difficult to control by crop management. A good example is the obligate biotrophic fungus *Blumeria graminis* DC. f. sp. *avenae* (Bga), the causal agent of the powdery mildew disease. Depending on the onset and course of infection powdery mildew can cause severe decreases in grain yield via reductions in fertile panicle numbers and grain weight (Roderick and Jones 1988; Clifford 1995).

The oat resistance gene *Pm7* was introduced into *Avena sativa* L. germplasm via the initial cross between *A. sativa* cv. ‘Solidor’ and *A. eriantha* (formerly named *A*. *pilosa*) accession CAV 0128, followed by embryo rescue for the initial cross and a series of backcrosses with oat cultivars (Kummer 1984). Hoppe (1991) selected several lines with resistance from CAV 0128 and provided the lines with the prefix APR, abbreviating ‘*A. pilosa* resistance’. For clarity, hereafter, APR is used as an abbreviation for the commonly used terminus adult plant resistance. Hoppe selected powdery mildew-resistant, euploid (2*n* = 42) progenies and conducted yield trials with the backcross-derived lines. The most promising lines were those with an *A. sativa* cytoplasm. Some of them even outyielded the recurrent parent ‘Solidor,’ but a yield gap of 30% relative to the more modern cultivar ‘Gramena,’ released in 1990, prevented their release as registered varieties. Hence, *Pm7* introgression lines were used in further backcrosses, and in 2008, 28 years after the initial cross with *A. eriantha,* the first cultivar ‘Canyon’ with *Pm7* resistance from CAV 0128 was released (https://www.bundessortenamt.de/apps9/web/bsa_sorteninfo/public/de). Plants carrying *Pm7* are easy to identify even under heavy powdery mildew infection pressure. This property makes the gene suitable for the use in oat breeding programs and, besides the already released cultivars with *Pm7*, a number of advanced breeding lines carrying this resistance gene are in the official testing pipelines. So far, *Pm7* has been used almost exclusively in European spring oat varieties. From 2010 to 2020, the share of spring oat varieties with *Pm7* resistance at the pan-European multiplication area for spring oats increased from 1 to roughly 9%. In 2020, twelve *Pm7*-carrying varieties covered more than 5600 ha of European multiplication area (Figure S1). In the UK AHDB field trial data between 2016 and 2020 (AHDB 2021), spring oat varieties with *Pm7* resistance showed a yield reduction of only 6% relative to fungicide-treated tests, whereas varieties lacking *Pm7* showed a yield reduction of 13% (Table S1). It may be expected that this advantage is even higher under high level of powdery mildew infection pressure. An increase in cultivation intensity and acreage of cultivars with the same single resistance gene bears the risk of the emergence of virulent races (Brown 2015). Therefore, it remains useful to identify new resistance sources to diversify the utilized resistance genes (Yu and Herrmann 2006; Sánchez-Martín et al. 2011; Herrmann and Mohler 2018; Ociepa et al. 2019). To use the sources of resistance more effectively, including the management of their deployment and their precise genome introgression, their mapping in the oat genome is necessary. Furthermore, the detailed genetic characterization of an effective resistance gene such as *Pm7* may provide clues for future molecular engineering of plant defense mechanisms.

Initial genetic studies of the resistance originating from CAV 0128 by Hoppe (1991) showed monogenic dominant inheritance, with an intermediate level of resistance on the first leaves and an increasing level with higher leaf insertions. This resistance continues to be effective at the adult plant stage across Europe with no known reports that the gene has been defeated.

Hsam et al. (2014) introduced the latest nomenclature for oat powdery mildew resistance genes and named the resistance locus *Pm7* which was transferred from *A. eriantha* to *A. sativa*. By using ‘Kanota’ monosomic line K9, Hsam et al. (2014) allocated the *Pm7* resistance locus to chromosome 13 according to the Jellen et al. (1997) nomenclature. Sanz et al. (2010), as well as Oliver et al. (2013), developed new nomenclatures with different approaches and maintained the designation of chromosome 13A based on monosomic line K9. The subsequent nomenclature by Chaffin et al. (2016) is referenced to Oliver et al. (2013), assigning chromosome 13A to Mrg12. Independent mapping experiments of the authors of this study regarding *Pm7* revealed a position on Mrg06, which contradicted the so far known map position and had to be clarified. At the meantime, complete genomes were sequenced (Waters 2020; Kamal et al. 2022) enabling a more precise positioning of *Pm7*.

In the present study, we have refined the chromosome location of *Pm7* to hexaploid oat Chromosome 5D considering the new nomenclature for the chromosomes of *Avena* species (https://wheat.pw.usda.gov/GG3/oatnomenclature) based on a genome-wide association study and mapping in two bi-parental populations segregating for *Pm7*.

## Material and methods

### Plant material

Three different resources were used for mapping of the *Pm7* resistance gene. The first was based on two oat diversity panels, namely KLAR1 and KLAR2, which together comprised 540 entries from 30 European oat breeding programs. The oat entries originate mainly from Germany, Austria, France, Poland, Hungary, Sweden and Slovakia (Table S2). In addition, two bi-parental populations, ‘Canyon’/‘Sam’ and AVA572/APR122, were developed at the University of Life Sciences in Lublin (Poland) and the Bavarian State Research Center for Agriculture (Germany), respectively. Crosses between the resistant cultivar ‘Canyon’ and the susceptible cultivar ‘Sam’ were followed by selfing the *F*_1_ and the *F*_2_ generations. *F*_3_ seeds were collected from 195 *F*_2_ individuals. Of these, 91 lines were selected randomly for genotyping experiments. The AVA572/APR122 mapping population was comprised of 90 *F*_2_-derived lines in the *F*_3_ generation as described by Hsam et al. (2014).

### Powdery mildew assessment and phenotypic data analysis

Two Bga isolates with different virulences were used to test at least 15 plants of each F_2:3_ line of the ‘Canyon’/‘Sam’ population. Detached leaf segment tests (LSTs) as described by Hsam et al. (2014) were carried out on the first leaves of 10-day-old seedlings. After 10 days of incubation, the results were scored and classified. Reactions to the isolates were grouped into two classes: resistant, with scores ranging from 0 to 20% infection relative to’Sam,’ and susceptible if the degree of infection exceeded 20%. The segregation ratio was determined using Chi-square tests of goodness of fit. The phenotypic data of the AVA572/APR122 population were established in the study of Hsam et al. (2014) with isolate H22 that was virulent to *Pm1*, *Pm2*, *Pm3*, *Pm6* and an undesignated gene in line 7718 Cn 20/3/1 but not virulent to *Pm4*, *Pm5*, *Pm7* and *Pm8*.

The two KLAR panels or subsets of them were grown from 2017 to 2020 in field trials located in Quedlinburg (QLB, 51° 47′ 31.402″ N 11° 8′ 29.213″ E, 140m asl, loam) and Groß Lüsewitz (GL, 54°04′ 15′′ N, 12°19′ 18′′ E; 37m asl, sandy loam). For each experiment, an 18 × 15 rectangular lattice design with two replications was used. A plot consisted of a double row of 1 m length and 20 cm distance between rows and plots. The severity of natural powdery mildew infection of the leaves was scored from 1 (no infection visible) to 9 (> 50% leaf coverage with powdery mildew pustules) during the plant developmental stages BBCH 61 to BBCH 75 (Hack et al. 1992). The KLAR1 panel was scored in 2017 in GL and QLB and the KLAR2 panel was scored in 2019 in QLB. In 2020, a selected subset of 252 lines of KLAR1 and KLAR2 was tested in GL and QLB. In the field experiments, there was no knowledge about prevalent Bga races, since there was only natural inoculation in each trial without an involvement of differential oat lines.

### Marker genotyping methods

For 92 F_2:3_ lines of the ‘Canyon’/‘Sam’ population, genomic DNA was extracted from 10-day-old bulked leaf samples of 15 plants using the DNeasy Plant Mini Kit (Qiagen). DNA preparation of the AVA572/APR122 population was described in Hsam et al. (2014). Genetic markers for ‘Canyon’/‘Sam’ and AVA572/APR122 were assayed using DArTseq, the genotyping-by-sequencing (GBS) service provided by Diversity Arrays Technology Pty Ltd (Bruce, Australia), which scores SNPs and presence/absence variants (PAVs or SilicoDArTs), as recently described (Sowa and Paczos-Grzęda 2020).

Genomic DNA of each entry of the diversity panel was isolated from fresh leaves according to the CTAB procedure described by Stein et al. (2001) and Tinker et al. (2009). For the diversity panel, GBS libraries were prepared by the Genomic Analysis Platform, Institute of Integrative Biology and Systems, Laval University (Québec, Canada) following the protocols described by Huang et al. (2014). The GBS libraries were subsequently sequenced by the McGill University and Génome Québec Innovation Centre (Montreal, Canada) using a HiSeq 2500 sequencer (Illumina, San Diego, CA).

### Genetic linkage mapping in ‘Canyon’/‘Sam’

For the ‘Canyon’/‘Sam’ population, the SilicoDArT and SNP data were filtered first in MS Excel for completeness (> 85%) and then manually curated to remove monomorphic and redundant markers. To avoid complications in positioning tightly linked dominant markers from opposite linkage phases (Mester et al. 2003; Hackauf et al. 2017), the SilicoDArT data were split into maternal and paternal data sets, depending on which parent contributed the dominant allele. For mapping of the *Pm7* gene, the respective resistance category of each *F*_2_ individual was added to the maternal, as well as paternal marker data set. Map construction using JoinMap®4.1 (Van Ooijen 2006) started with 814 paternal loci and 447 maternal loci. Both datasets were mapped as *F*_2_ comprising each 91 individuals. Identical and similar (0.97) loci were excluded, and independence logarithm of the odds ratio (LOD) for grouping, and regression mapping using Kosambi’s function for mapping were applied. By adding codominant loci of the constructed maternal map to the paternal map and vice versa, the “extended” maternal and paternal maps were created and these two were joined via "combine groups for map integration" in the "Join" menu. DArT Marker sequences were aligned to the genome sequence assembly of *A. sativa* cv. OT3098 available on GrainGenes (https://wheat.pw.usda.gov/GG3/graingenes_downloads/oat-ot3098-pepsico, Blake et al. 2019) using BWA-MEM (Li 2013). Primary alignments were extracted with SAMtools (Li et al. 2009).

### Genetic linkage mapping in AVA572/APR122

For the AVA572/APR122 population, markers were curated using the R package synbreed V0.12-6 (Wimmer et al. 2012). GBS-derived SNP markers were discarded when the parental genotypes were unavailable or monomorphic. Only non-redundant markers showing < 10% missing values, a minor allele frequency > 0.05 and a regular segregation ratio were retained for further analysis. For genetic map construction, markers that were either mapped in the oat consensus map (Chaffin et al. 2016) or located on the OT3098 hexaploid oat pseudomolecules (https://triticeaetoolbox.org/oat/) were used preferentially. Six dominant amplified fragment length polymorphism (AFLP) markers and the qualitative phenotype scores from the study of Hsam et al. (2014) were added to the GBS data. Map construction followed methods similar to the ‘Canyon’/‘Sam’ population using separate maternal and paternal data sets, except that JoinMap® software version 5.0 (Kyazma BV, Wageningen, The Netherlands) was used. Linkage group construction was established at LOD ≥ 3.0, with a maximum recombination fraction of 0.4, and regression mapping was performed using the Haldane mapping function. Genetic linkage maps were drawn with Mapchart 2.1 software (Voorrips 2002).

### Genotypic data analysis, statistical analysis of powdery mildew data and GWAS

Deconvolution of raw sequence data of the KLAR diversity panels was performed using the UNEAK pipeline described by Lu et al. (2013), resulting in a file of GBS tag counts for each sequenced DNA sample. Subsequent GBS-SNP calling was performed using the software ‘Haplotag’ (Tinker et al. 2016) in the production mode based on a GBS nomenclature and methods described by Bekele et al. (2018). The resulting Hapmap file was filtered for 90% minimum count and minor allele frequency of 0.05, resulting in a set of 8012 GBS–SNP markers. The filtered file was merged with marker positions on the OT3098 hexaploid oat genome downloaded from T3/Oat (https://triticeaetoolbox.org/oat/). Missing data were then imputed using the function LinkImpute (Money et al. 2015) (parameters: high LD sites = 30, nearest neighbors = 10, max distance between site to find LD = 10.000.000) implemented in TASSEL version 5.2.66 (Bradbury et al. 2007).

The underlying population structure was investigated using TASSEL by conducting a principal component analysis (PCA) and by calculating a kinship matrix (K) using Centered IBS option. Five PCs and K were included into the mixed-linear model (MLM; without compression, but P3D) (Zhang et al. 2010) in TASSEL utilized for the association analysis. The threshold for significance of MTAs was calculated according to Benjamini and Hochberg (1995) with a false discovery rate (FDR) of *p* = 0.05. This association analysis protocol including a set of 8012 markers was applied to nursery data of two population subsets each comprising 232 entries grown in Quedlinburg and Groß Lüsewitz in 2017 and 2020, respectively. To homogenize error variances, the powdery mildew score data were Box–Cox transformed (Box and Cox 1964). With data of 116 entries, orthogonal for the two years and sites, we applied a linear mixed-effects model using r package lme4 (Bates et al. 2015) and the lmer function to estimate variance components for the random factors oat line (*G*), site (*L*), year (*Y*), replication (REP) and the two- and threefold interactions of *G*, *L* and *Y*. The variance components were used to calculate broad sense heritability according to equation with *l*, *y*, and *r* as number of locations, years and replications, respectively.

$${H}^{2}={\sigma }_{G}^{2}/{[\sigma }_{G}^{2}+{(\sigma }_{GL}^{2}/\mathrm{l})+{(\sigma }_{GY}^{2}/\mathrm{y})+{(\sigma }_{GLY}^{2}/ly)+({\sigma }_{R}^{2}/rly)]$$

## Results

### Linkage mapping in the ‘Canyon’/‘Sam’ population

The detached leaf assays based on two different Bga isolates enabled a clear grouping of the individuals of the ‘Canyon’/‘Sam’ population into three categories: homozygous resistant, susceptible, or segregating. The segregation ratio in both tests was the same, including 47 resistant: 92 segregating: 56 susceptible, which fit the expected 1:2:1 ratio of a single dominant gene (*χ*^2^ = 1.34; *p* value = 0.512).

**Fig. 1:**
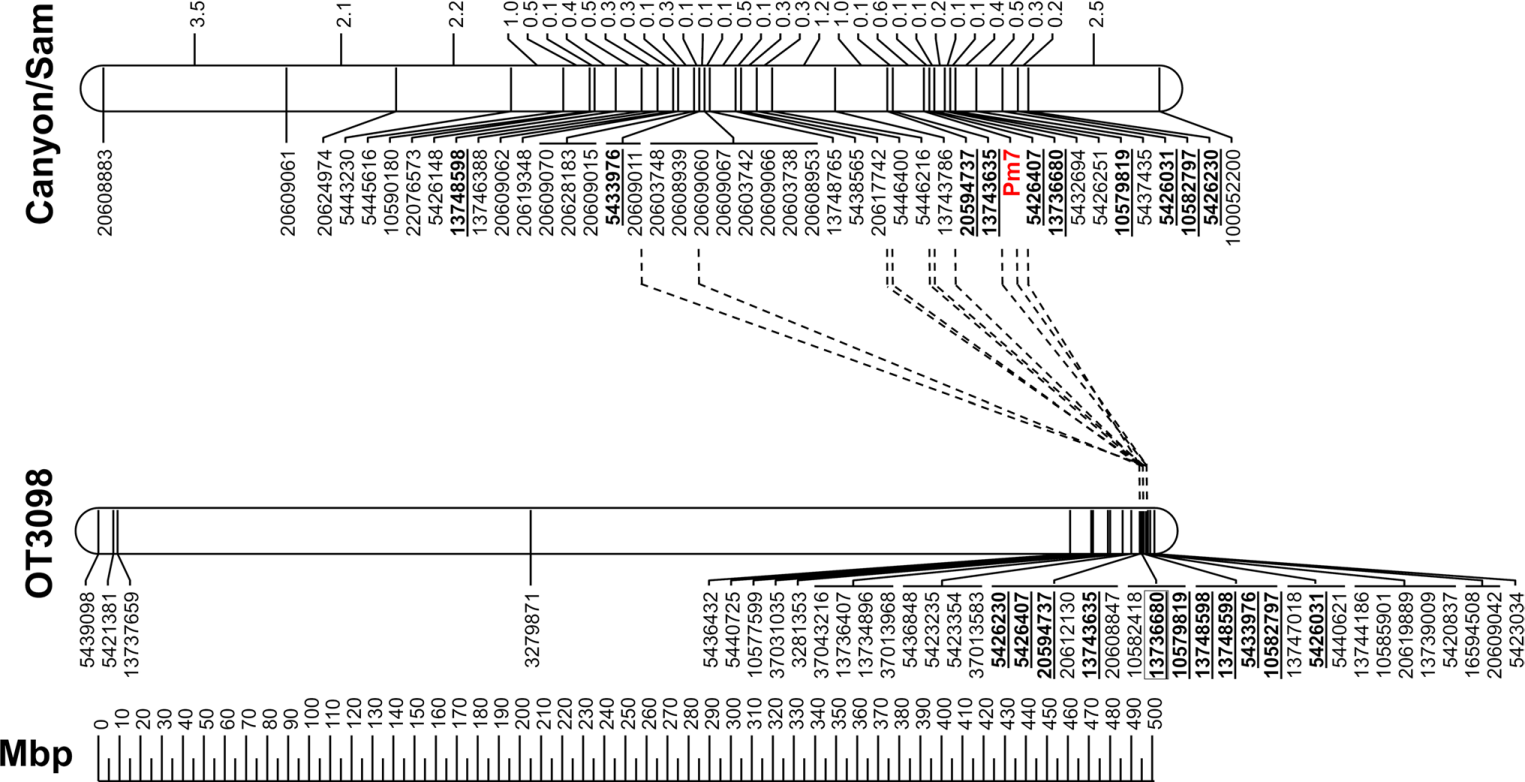
Comparison of ‘Canyon’/‘Sam’ genetic map of 5D with the *Pm7* gene and the physical map of 5D of the OT3098 genome

Mapping of *Pm7* in the paternal map resulted in a linkage group with 24 markers and a length of 15.1 cM. The maternal map comprised 34 markers covering 21.4 cM. After joining both maps, the resulting ‘Canyon’/‘Sam’-map consists of 44 markers and 20.2 cM length. The order of these markers was compared to a map of chromosome 5D markers which was developed by BLASTing DArT sequences to the OT3098 V.1 sequence. The outcome was an assignment of the ‘Canyon’/‘Sam’ genetic map to the distal end of the long arm of chromosome 5D near a cluster of markers at 494,888,790 bp (Fig. 1).

### Linkage mapping in the AVA572/APR122 population

**Fig. 2:**
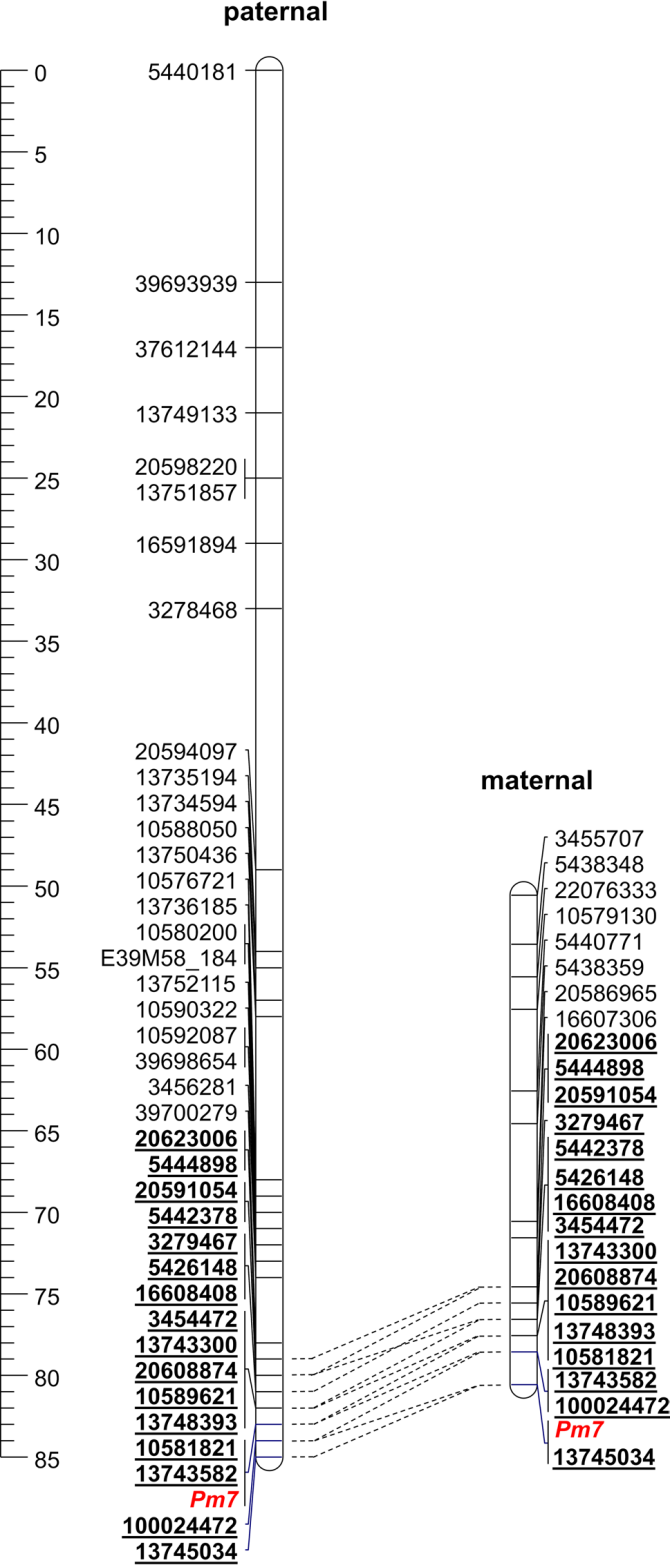
Paternal map vs. maternal map of chromosome 5D based on linkage mapping in AVA572/APR122 population. Common loci are underlined. For reasons of clarity, parent-specific markers are not shown for the *Pm7* target region

Of 33,386 dominant DArT markers, 3,586 remained after marker pruning in the AVA572/APR122 population. DArT markers were discarded due to missing polymorphism between parents (37%) or missing genotyping values (22%). Four and two AFLP markers from the study of Hsam et al. (2014) were added to the paternal and maternal data set, respectively. Of 11,455 SNP markers, 6604 conformed to segregation patterns expected for oats. The codominant markers together with the powdery mildew genotypes from the study of Hsam et al. (2014) were added to each of the two parent-specific marker data files. The paternal genetic map including the *Pm7* resistance gene had a length of 86 cM and comprised 52 molecular markers including 16 codominant SNP, two dominant SNP, 30 DArT and 4 AFLP markers (Table S3). Ten uncharacterized markers were used to bridge the map distance between DArT markers 5,440,181 and 13,750,436 representing the most distal markers on the consensus genetic and the physical map, respectively. The maternal genetic map contained 38 molecular markers and spanned a map distance of 30 cM (Table S3). The order of the codominant SNP markers constituting the two genetic maps was sufficiently similar (Fig. 2). In both maps, the closest codominant SNP markers to *Pm7* were 13,743,582, 100,024,472 and 13,745,034 spanning a physical distance of 20 Mb. Both maps suggest a location of *Pm7* at the distal end of the long arm of chromosome 5D.

### Pm resistance in the KLAR panels and GWAS

The powdery mildew infection in the KLAR panels showed highly reproducible differences among the tested oat varieties over years and locations, indicated by a broad sense heritability of 0.96 for the orthogonal tested subset of lines over both years and locations. Specifically, oat varieties with known resistance genes according to pedigree and previous experiments were found to be resistant (Table S4) including those with genes *Pm7* (28 lines), *Pm5* (5 lines; Yu and Herrmann 2006), an uncatalogued gene with resistance from *A. strigosa*_AVE488 (5 lines, tentative gene designation *Pm488*) and one line with resistance from *A. occidentalis* CAV3889 (tentative gene designation *Pm3889*; Herrmann and Roderick 1996). In addition, several lines were found to possess unknown resistance genes. The incomplete, adult plant resistances known for cultivars ‘Husky,’ ‘Keely,’ ‘Auteuil,’ ‘Firth,’ ˈFlämingstip’ and others were also observed (Table S4).

Marker pruning of the 250 k GBS–SNP sequences of the panel resulted in a set of 8012 GBS–SNP markers. The density and distribution of pruned markers over the genome are displayed in Figure S2. Most regions of the genome were evenly covered by GBS markers with the exception of several large gaps in more medial regions on chromosomes 1A, 2A, 2C, 3C, 3D, 4A, 4D, 5C, 6C, 6D and 7A (Figure S2). To explore the underlying population structure of the oat panel, five principal components were calculated, of which the first three principal components explained 10.6%, 7.7%, and 4.9% of the genetic variation, respectively. Scatterplots of the first three principal components (Figures S3–S5) showed a moderate clustering of the panel. Varieties with the *Pm7* gene belong to different main clusters but do not occur in all main clusters.

The two GWAS runs, each with 232 oat lines (116 in common), identified different numbers of significant MTAs (Table 1). The GWAS with 2017 data resulted in five markers on chromosome 5D and one marker on chromosome 4A. In the GWAS with 2020 data, 13 markers on chromosome 5D, including the five markers of the GWAS 2017 run, plus two markers on chromosomes 3C and 1A, respectively, were found. Two more GWAS analyses, based only on the *Pm7* carrier *vs.* susceptible lines, yielded MTAs only on chromosome 5D (data not shown for brevity). All associated markers on chromosome 5D are located at the distal end between the base pairs 473,055,514 and 501,576,522 (Table 1). The GWAS 2017 and 2020 results are visualized in Manhattan plots in Figures S6 and S7.

**Fig. 3:**
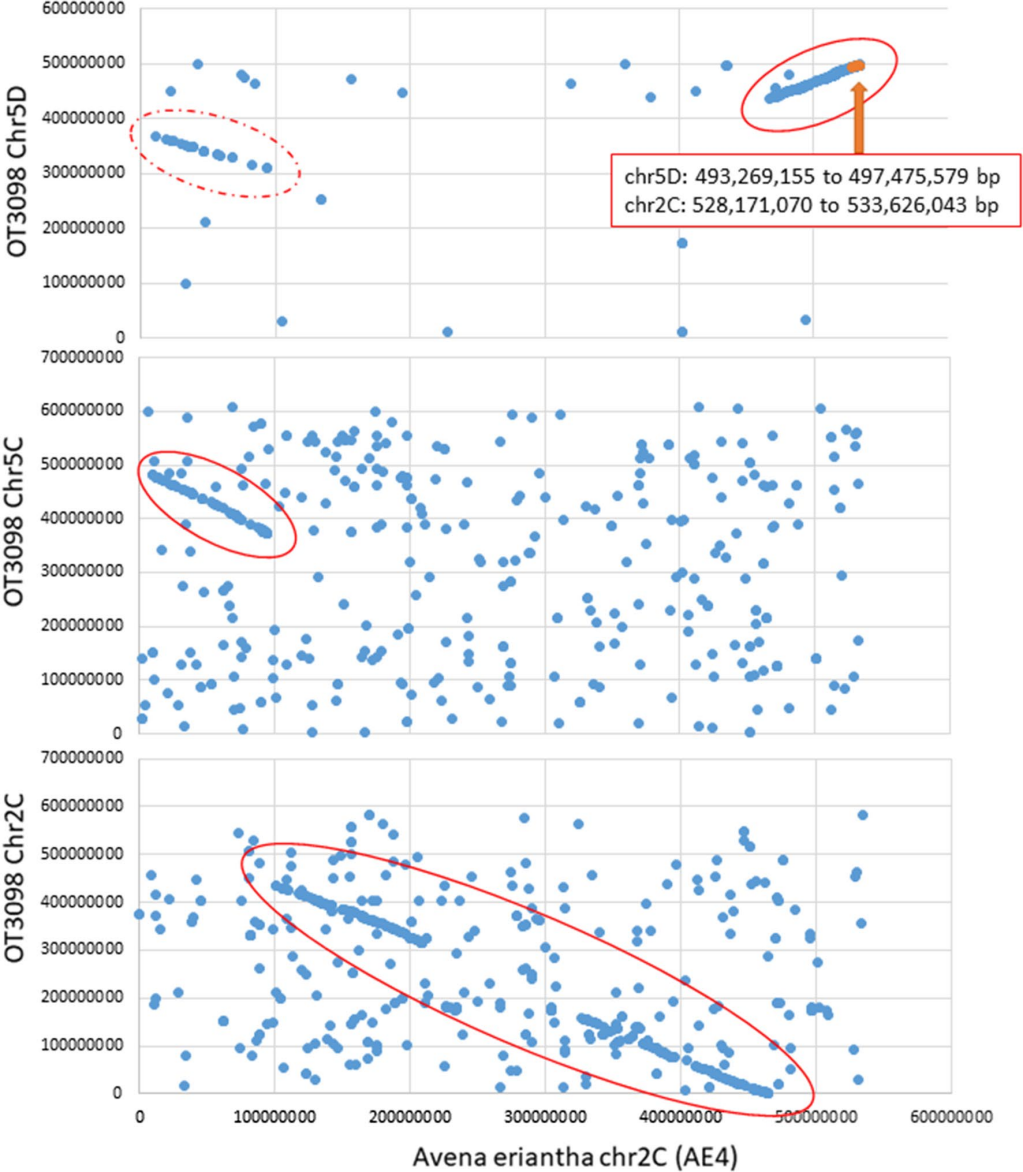
Collinear regions in diploid vs hexaploid oat (OT3098). Blue dots show joint coordinates of perfect BLAST matches of GBS alleles in hexaploid *A. sativa* OT3098 versus *A. eriantha* CN 19,328. Solid red circles show inferred linear homologous regions. Matches outside of these regions are likely to be spurious, thus there are a higher density of spurious matches on C-genome versus D-genome comparisons. The homologous region on chromosome 2C is broken at the centromeres due to a lack of GBS matches in these highly repetitive regions. The linear match in the dotted red circle on chromosome 5D is likely a “shadow” caused by homeologous matches. The orange arrow indicates GBS loci and bp positions associated with *Pm7*

**Fig. 4:**
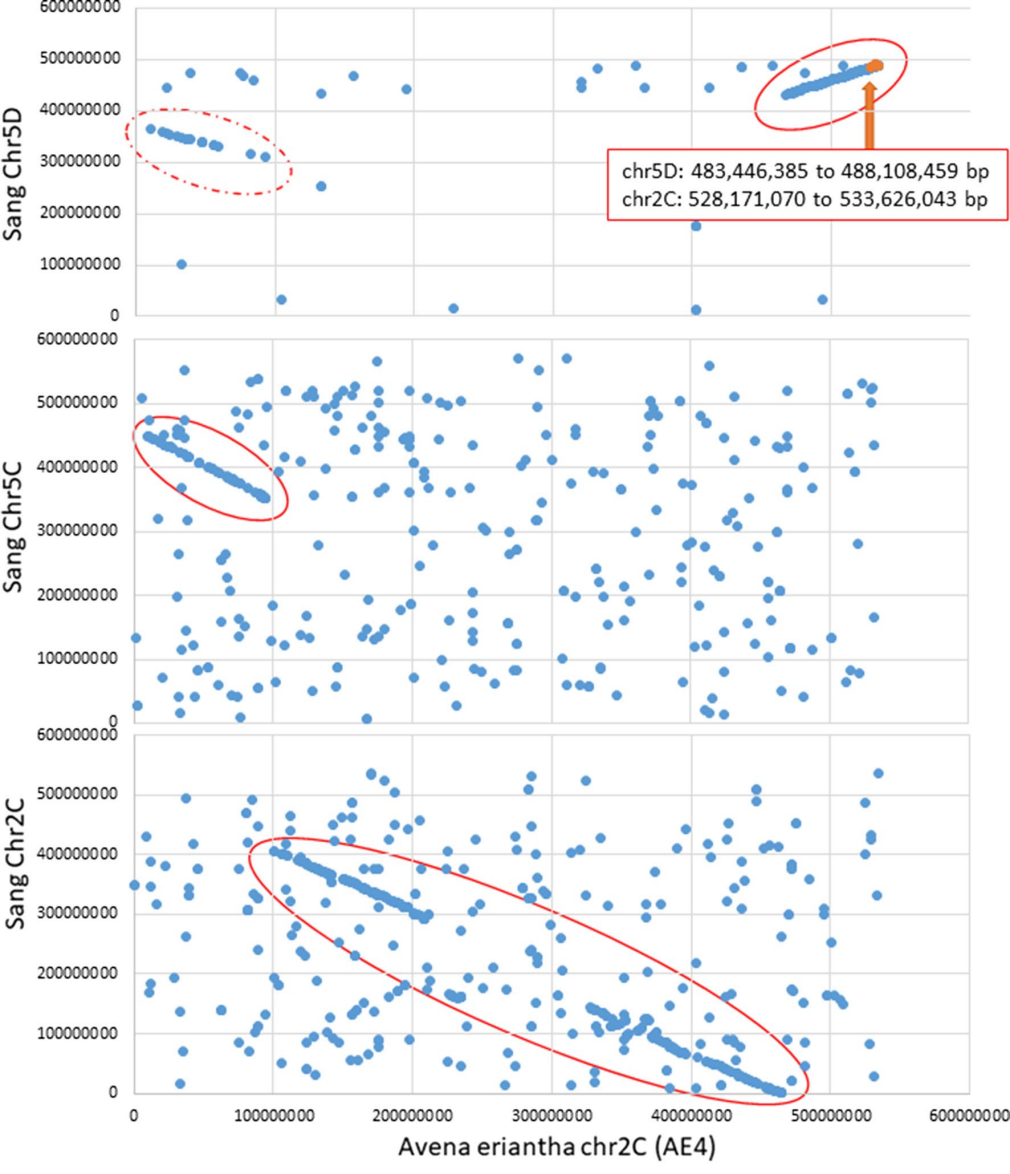
Collinear regions in diploid vs hexaploid oat (cv. Sang). Blue dots show joint coordinates of perfect BLAST matches of GBS alleles in hexaploid *A. sativa* Sang vs. *A. eriantha* CN 19,328. Solid red circles show inferred linear homologous regions. Matches outside of these regions are likely to be spurious, thus there are a higher density of spurious matches on C-genome vs D-genome comparisons. The homologous region on chromosome 2C is broken at the centromeres due to a lack of GBS matches in these highly repetitive regions. The linear match in the dotted red circle on chromosome 5D is likely a “shadow” caused by homeologous matches. The orange arrow indicates GBS loci and bp positions associated with *Pm7*

A local alignment (Table S5) of the sequences of the significant GBS markers with the *A. eriantha* genome sequence showed that the majority of matches were located on the distal end of chromosome AE4 between 528,171,070 and 533,626,043 bp. Dot plot comparisons (Figs. 3 and 4) showed linear homologous matches between *A. eriantha* chromosome AE4 and *A. sativa* OT3098 and ‘Sang’ for chromosomes 2C, 5C, and 5D suggesting that the common C-genome ancestor of *A. sativa* has been fragmented at least twice, including an intergenomic translocation to chromosome 5D placing the *Pm7* location at the distal end of chromosome 5D in hexaploid oat.

### Candidate genes for Pm7

We parsed the gene annotations of the *Pm7* region in the genomes of OT3098, ‘Sang’ and *A. eriantha* for resistance related gene products “kinase” and “LRR,” known to be a crucial part of plant immunity and pathogen perception (Schultink and Steinbrenner 2021). Most frequently found genes were those coding for lectin S-receptor-like serine/threonine kinases (19 hits) and cysteine-rich receptor-like kinases (17 hits) (Table S6). In addition, we got nine hits for nucleotide-binding domain and leucine-rich repeat (NLR) immune receptors and nucleotide-binding domain shared with APAF-1, various R-proteins and CED-4 (NB-ARC), the central switches in regulating NLR activity (Steele et al. 2019; Wang et al. 2020).

## Discussion

### Pm7 usage

*Pm7* is currently one of the most effective genes against powdery mildew in oats (Okoń 2015). The utilization of this resistance is simplified by the complete absence of virulent races and its effectiveness from young to adult plant stages. In LSTs with the first or second leaf of plants carrying *Pm7*, there is some pustule growth and weak sporulation (Okoń and Ociepa 2017). Hoppe (1991) also observed weaker resistance of first leaves and increasing resistance of higher leaf insertions in APR122, the *Pm7* donor line for the oat materials of this study. Oat cultivars with the *Pm7* gene respond differently to infection with Bga. For example, Bga virulence studies conducted in recent years have shown that the variety ‘Canyon’ exhibits a different infection profile in response to pathogen attack than the ARP122 line used as a control line in many studies of this type (Okoń et al. 2021; Cieplak et al. 2022). This may suggest a new allelic form of the *Pm7* gene, which could be caused by recombination within the introduced segment leading to some susceptibility to races that are not virulent for *Pm7* in APR122. However, changes in virulence profiles do not negate the highly effective resistance conferred by *Pm7*.

### Map position of Pm7

Linkage mapping with two independent populations located the *Pm7* gene to the distal end of chromosome 5D. The imperfect correspondence between the marker order of the genetic maps and to the physical map is at least partly related to the small population size of the two populations. It is well known that genetic maps based on dominant markers in small populations are prone to inconsistencies in marker order (Ferreira et al. 2006). However, in the GWAS runs including *Pm7* carriers, the strongest MTAs were also located in the terminal region of chromosome 5D, providing strong evidence for a consistent and stable location of *Pm7*.

Given this strong evidence for a location on chromosome 5D, the previous assignment of *Pm7* to chromosome 13A (chromosome 7A) requires revision. The initial assignment by Hsam et al. (2014) was based on a deviation from the expected segregation ratio of the cross that involved line K9, which is monosomic for chromosome 13A and derived from *Avena byzantina* cv. ‘Kanota’ (Morikawa 1985). However, aneuploid chromosome assignments often suffer from artifacts (Singh and Kolb 1991) and various explanations for this incorrect assignment are possible, including the existence of novel chromosome rearrangements in the aneuploid lines. There are many reports about chromosomal rearrangements (Chen and Armstrong 1994, Jellen et al. 1994, Leggett and Markhand 1995, Sanz et al. 2010, Chaffin et al. 2016) which led to a mosaic-like genome architecture (Kamal et al. 2022).

Genotyping and phenotyping the KLAR panel offered the opportunity to genetically map other resistance genes besides *Pm7*, such as *Pm5*, *Pm488*, *Pm3889*, and unknown resistance genes. Since *Pm7* was mapped on 5D, the MTAs on 4A (2017 data GWAS), 1A and 3C (2020 data GWAS) are expected to be associated with other resistance genes. These three MTAs are only significant for an FDR of 0.05 and not for *p* = 0.01. The knowledge that other resistance genes are present in the panel besides *Pm7* leads to the question of why there were not more, or stronger marker-trait associations. The identification of MTAs depends on the FDR setting, marker density and coverage, population size and structure, quality of phenotypic data, and allele frequency. In the GWAS 2017 panel, there are five oat lines with the *Pm5* gene, and in the 2020 panel, there is only one line, resulting in a weak MTA on chromosome 4A only for the GWAS 2017. Another resistance gene, effective in Firth and possibly more cultivars, mapped on chromosome 1D (Hagmann et al. 2012), was apparently present in the panel, but no MTA was found in the present calculations. For the resistance genes *Pm488* and *Pm3889*, the five (*Pm488*) and one (*Pm3889*) lines in the panel are probably too infrequent to find unambiguous MTAs. In addition to this low allele frequency, it is also possible that gaps or low marker coverage have prevented identification of MTAs.

The GWAS with the 2020 panel data detected MTAs on 1A and 3C, indicating the presence of *Pm3* and *Pm6*, respectively, in the panel (Table S7). This is explainable, as both *Pm3* and *Pm6*, representing former OMR 1 and 3, respectively, are present in many former varieties (Hsam et al. 1997), which in turn may have served as parents for some of the lines of the KLAR panel. However, since the resistance genes of the KLAR panel have not been determined by using defined differentiating mildew isolates, an involvement of *Pm3* and *Pm6* within the panel remains uncertain.

### Candidate genes for Pm7

A preliminary screening for candidate genes showed that the region of chromosome 2Ce in *A. eriantha*, as well as the corresponding region of chromosome 5D in the hexaploid genomes, contains a high frequency of genes annotated as leucine-rich repeat domain receptor-like kinases (LRR-RK) or NLRs class in the *Pm7*-region of three plant genomes of OT3098, ‘Sang’ and *A.* *eriantha.* The most frequently found lectin S-receptor-like serine/threonine kinases are involved in resistances in many plant pathogen systems (Cao et al. 2011; Sánchez-Martín et al. 2021). The second most frequently found proteins were cysteine-rich receptor-like kinases (17 hits), which, for example, provides a broad-spectrum resistance to *Septoria tritici* blotch (Saintenac et al. 2021).

The NLRs are intracellular receptor gene families in plants and animals as well recognizing effectors of pathogens and initiate rapid defense responses (Li et al. 2019; Sánchez-Martín and Keller 2021). There are some more functions of NLRs and a large diversity of NLR genes, of which about 100 have been cloned (Kapos et al. 2019). Results of this study do not show, if the *Pm7* gene is related to any variant within the detected genes coding for NLRs or LRR-RK in the 5D region. Future efforts with *Pm7* carrier are needed to fine map and clone the Pm7 gene.

The positioning of *Pm7* on chromosome 5D is interesting, since *A. eriantha* carries the C-genome, while the D-genome is highly related to A-genome diploids. However, Maughan et al. (2019) showed that *A*. *eriantha* chromosomes AE4, AE5 and AE7 all share significant numbers of D-genome markers in addition to the expected C-genome markers, confirming earlier predictions that C/D-genome translocations are a common occurrence in hexaploid oat (Yan et al. 2016). In dot-plot-based reference genome comparisons, a C-to-D-genome translocated region can now be seen clearly on the terminal region of chromosome 5D (Figs. 3 and 4). Further genome sequencing will be required to confirm whether all or some hexaploid oats contain this translocated region. However, the presence of this translocated region suggests a probable reason for the integration of *Pm7* from the *A. eriantha* donor through partial homologous pairing with a terminal chiasma resulting in recovery of a balanced chromosome 5D gamete. More precise comparisons of the introgression in *A. sativa* and the donor region in *A. eriantha* will require further fine mapping and sequencing of the *Pm7* gene from the donor and a resistant cultivar.

In oat breeding, practical selection of plants carrying *Pm7* is easy as long as powdery mildew is present, which is not always the case. Therefore, sequence information of linked markers from this and ongoing studies may be used to develop KASP assays for the more efficient selection of resistant plants carrying *Pm7*.

**Table 1:**
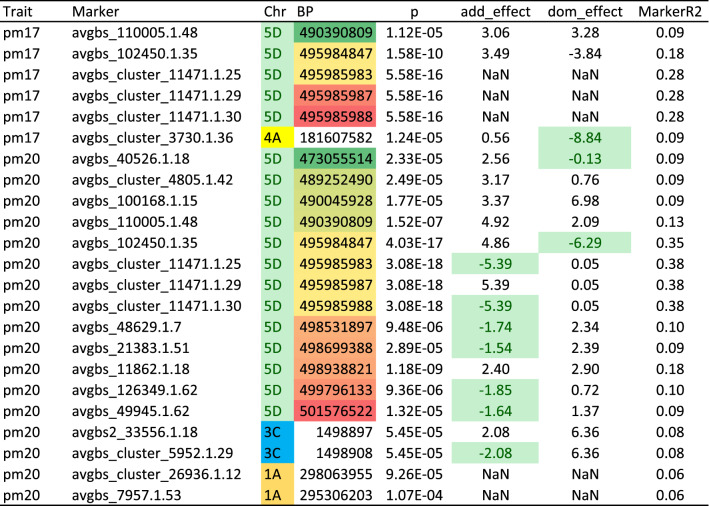
Significant (*p* = 0.05) marker-trait associations for the GWAS runs with powdery mildew 2017 (pm17) and 2020 (pm20) data, the chromosomal position (BP), the additive and dominance effects of markers and Marker R2 values, based on BoxCox-transformed data (color table online)

## Supplementary Information

Below is the link to the electronic supplementary material.

Supplementary file1 (DOCX 54 KB)

Supplementary file2 (JPG 2697 KB)

Supplementary file3 (TIFF 769 KB)

Supplementary file4 (TIFF 769 KB)

Supplementary file5 (TIFF 769 KB)

Supplementary file6 (TIFF 676 KB)

Supplementary file7 (TIFF 676 KB)

Supplementary file8 (DOCX 14 KB)

Supplementary file9 (XLSX 32 KB)

Supplementary file10 (XLSX 17 KB)

Supplementary file11 (XLSX 162 KB)

Supplementary file12 (XLSX 21 KB)

Supplementary file13 (XLSX 29 KB)

Supplementary file14 (XLSX 14 KB)

## Data Availability

The datasets generated during and/or analyzed during the current study are available from the corresponding author on reasonable request.

## Abbreviations

AFLP: Amplified fragment length polymorphism
APRu: Adult plant resistance by unknown resistance genes
Bga: *Blumeria graminis* DC. f. sp. *avenae*
FDR: False discovery rate
GBS: Genotyping-by-sequencing
GL: Groß Lüsewitz
GWAS: Genome-wide association study
K: Kinship matrix
LOD: Logarithm of the odds ratio
LST: Detached leaf segment tests
MLM: Mixed linear model
MTA: Marker trait association
PAVs: Present/absence variants
PCA: Principal component analysis
QLB: Quedlinburg
SNP: Single nucleotide polymorphism

## References

[CR1] AHDB (2021) Recommended Lists for cereals and oilseeds (RL) harvest results (archive) (UK), https://ahdb.org.uk/knowledge-library/recommended-lists-for-cereals-and-oilseeds-rl-harvest-results-archive

[CR2] Ahola HG, Sontag-Strohm TS, Schulman AH, Tanhuanpää P, Viitala S, Huang X (2020). Imunochemical analysis of oat avenins in an oat cultivar and landrace collection. J Cereal Sci.

[CR3] Bates D, Mächler M, Bolker B, Walker S (2015) Fitting linear mixed-effects models using lme4. J Stat Softw 67:1–48

[CR4] Bekele WA, Wight CP, Chao S, Howarth CJ, Tinker NA (2018). Haplotype-based genotyping-by-sequencing in oat genome research. Plant Biotechnol J.

[CR5] Benjamini Y, Hochberg Y (1995). Controlling the false discovery rate: a practical and powerful approach to multiple testing. J R Stat Soc B.

[CR6] Blake VC, Woodhouse MR, Lazo GR, Odell SG, Wight CP, Tinker NA, Wang Y, Gu YQ, Birkett CL, Jannink J-L, Matthews DE, Hane DL, Michel SL, Yao E, Sen TZ (2019). GrainGenes: centralized small grain resources and digital platform for geneticists and breeders. Database.

[CR7] Box GEP, Cox DR (1964). The analysis of transformations (with discussion). J R Stat Soc B.

[CR8] Bradbury PJ, Zhang Z, Kroon DE, Casstevens TM, Ramdoss Y, Buckler ES (2007). TASSEL: software for association mapping of complex traits in diverse samples. Bioinformatics.

[CR9] Brown JKM (2015). Durable resistance of crops to disease: a Darwinian perspective. Annu Rev Phytopathol.

[CR10] Cao A, Xing L, Wang X, Yang X, Wang W (2011). Serine/threonine kinase gene *Stpk-V*, a key member of powdery mildew resistance gene *Pm21*, confers powdery mildew resistance in wheat. PNAS.

[CR11] Chaffin AS, Huang YF, Smith S, Bekele WA, Babiker E, Gnanesh BN, Foresman BJ, Blanchard SG, Jay JJ, Reid RW, Wight CP, Chao S, Oliver R, Islamovic E, Kolb FL, McCartney C, Mitchell Fetch JW, Beattie AD, Bjørnstad Å, Bonman JM, Langdon T, Howarth CJ, Brouwer CR, Jellen EN, Klos KE, Poland JA, Hseih TF, Brown R, Jackson E, Schlueter JA, Tinker NA (2016). A consensus map in cultivated hexaploid oat reveals conserved grass synteny with substantial subgenome rearrangement. Plant Genome.

[CR12] Chen Q, Armstrong K (1994). Genomic in situ hybridization in *Avena sativa*. Genome.

[CR13] Cieplak M, Nucia A, Ociepa T, Okon S (2022). Virulence structure and genetic diversity of *Blumeria graminis* f. sp. *avenae* from different regions of Europe. Plants.

[CR14] Clifford BC, Welch RW (1995). Diseases, pests and disorders of oat. The oat crop.

[CR15] Ferreira A, da Silva MF, Cruz CD (2006). Estimating the effects of population size and type on the accuracy of genetic maps. Genet Mol Biol.

[CR16] Hack H, Bleiholder H, Buhr L, Meier U, Schnock-Fricke U, Weber E, Witzenberger A (1992). A uniform code for phenological growth stages of mono- and dicotyledonous plants—extended BBCH scale, general. Allg Nachr Deutsch Pflanzenschutzd.

[CR17] Hackauf B, Haffke S, Fromme FJ, Roux SR, Kusterer B, Musmann D, Kilian A, Miedaner T (2017). QTL mapping and comparative genome analysis of agronomic traits including grain yield in winter rye. Theor Appl Genet.

[CR18] Hagmann E, von Post L, von Post R, Eklund M, Larsson CT, Tuvesson S, Vallenback P, Ceplitis A (2012) QTL mapping of powdery mildew resistance in oats using DArT markers. In: Proceedings of 15th international EWAC conference, 7–11 Nov 2011, Novi Sad, Serbia, European Cereals Genetics Co-operative Newsletter, pp 91–94

[CR19] Herrmann MH, Mohler V (2018). Locating two novel genes for resistance to powdery mildew from *Avena byzantina* in the oat genome. Plant Breed.

[CR20] Herrmann MH, Roderick HW (1996). Characterisation of new oat germplasm for resistance to powdery mildew. Euphytica.

[CR21] Herrmann MH, Schmehe B, Brodführer S (2020) Kleistogamer Hafer zur nachhaltigen Vermeidung von Flugbrand (KLAR). Schlussbericht FKZ_2815NA107 [cleistogamic oats for a sustainable smut prevention]. https://orgprints.org/id/eprint/38456/1/Schlussbericht_gesamt.pdf

[CR22] Hoppe HD (1991) Möglichkeiten zur Introgression von wildartinformation aus *Avena*-Arten niederer Ploidiestufen und deren züchterische Nutzung in hexaploidem Kulturhafer Diss, Martin-Luther-Universität Halle-Wittenberg, p 90

[CR23] Hsam SLK, Mohler V, Zeller FJ (2014). The genetics of resistance to powdery mildew in cultivated oats (*Avena sativa* L.): current status of major genes. J Appl Genet.

[CR24] Hsam SLK, Peters N, Paderina EV, Felsenstein F, Oppitz K, Zeller FJ (1997). Genetic studies of powdery mildew resistance in common oat (*Avena sativa* L.) I cultivars and breeding lines grown in Western Europe and North America. Euphytica.

[CR25] Huang YF, Poland JA, Wight CP, Jackson EW, Tinker NA (2014). Using genotyping-by-sequencing (GBS) for genomic discovery in cultivated oat. PLoS ONE.

[CR26] Jellen EN, Gill B, Cox T (1994). Genomic in situ hybridization differentiates between A/D-and C-genome chromatin and detects intergenomic translocations in polyploid oat species (genus *Avena*). Genome.

[CR27] Jellen EN, Rines HW, Fox SL, Davis DW, Phillips RL, Gill BS (1997). Characterization of ‘Sun II’ oat monosomics through C-banding and identification of eight new ‘Sun II’ monosomics. Theor Appl Genet.

[CR28] Kamal N, Tsardakas Renhuldt N, Bentzer J, Gundlach H, Haberer G, Juhasj A, Lux T, Bose U, Tye-Din J, Lang D, van Gessel N, Reski R, Fu Y-B, Spégel P, Ceplitis A, Himmelbach A, Waters AJ, Bekele WA, Colgrave M, Hansson M, Stein N, Mayer K, Jellen EN, Maughan PJ, Tinker NA, Mascher M, Olsson O, Spannagl M, Sirijovski N (2022). The mosaic oat genome gives insights into a uniquely healthy cereal crop. Nature.

[CR29] Kapos P, Devendrakumar KT, Li X (2019). Plant NLRs: from discovery to application. Plant Sci.

[CR30] Kummer M (1984). Neue resistenzquellen bei echtem mehltau für die züchtung des saathafers. Tag-Ber Akad Landwirtsch-Wiss Berlin.

[CR31] Leggett JM, Markhand GS (1995). The genomic identification of some monosomics of *Avena sativa* L. cv. Sun II using genomic in situ hybridization. Genome.

[CR34] Li H (2013) Aligning sequence reads, clone sequences and assembly contigs with BWA-MEM. arXiv:1303.3997v2

[CR32] Li H, Handsaker B, Wysoker A, Fennell T, Ruan J, Homer N, Marth G, Abecasis G, Durbin R (2009). The sequence alignment/map format and SAMtools. Bioinformatics.

[CR33] Li Z, Huang J, Wang Z, Meng F, Zhang S, Wu X, Zhang Z, Gao Z (2019). Overexpression of arabidopsis nucleotide-binding and leucine-rich repeat genes RPS2 and RPM1(D505V) confers broad-spectrum disease resistance in rice. Front Plant Sci.

[CR35] Lu F, Lipka AE, Glaubitz J, Elshire R, Cherney JH, Casler MD (2013). Switchgrass genomic diversity, ploidy, and evolution: novel insights from a network-based SNP discovery protocol. PLoS Genet.

[CR36] Maughan PJ, Lee R, Walstead R, Vickerstaff RJ, Fogarty MC, Brouwer CR, Reid RR, Jay JJ, Bekele WA, Jackson EW, Tinker NA, Langdon T, Schlueter JA, Jellen EN (2019). Genomic insights from the first chromosome-scale assemblies of oat (*Avena* spp) diploid species. BMC Biol.

[CR37] Mester DI, Ronin YI, Hu Y, Peng J, Nevo E, Korol AB (2003). Efficient multipoint mapping: making use of dominant repulsion-phase markers. Theor Appl Genet.

[CR38] Money D, Gardner K, Migicovsky Z, Schwaninger H, Zhong GY, Myles S (2015). LinkImpute: fast and accurate genotype imputation for nonmodel organisms. G3 Genes Genomes Genet.

[CR39] Morikawa T (1985). Identification of the 21 monosomic lines in *Avena byzantina* C Koch cv ‘Kanota’. Theor Appl Genet.

[CR40] Ociepa T, Okoń SM, Nucia A, Leśniowska-Nowak J, Paczos-Grzęda E, Bisaga M (2019). Molecular identification and chromosomal localization of new powdery mildew resistance gene Pm11 in oat. Theor Appl Genet.

[CR41] Okoń SM (2015). Effectiveness of resistance genes to powdery mildew in oat. Crop Prot.

[CR42] Okoń SM, Ociepa T (2017). Virulence structure of the *Blumeria graminis* DCf sp. *avenae* populations occurring in Poland across 2010–2013. Eur J Plant Pathol.

[CR43] Okoń SM, Cieplak M, Kuzdralinski A, Ociepa T (2021) New pathotype nomenclature for better characterization the virulence and diversity of *Blumeria g*raminis f.sp. *avenae* populations. Agronomy. 11:1852

[CR44] Oliver RE, Tinker NA, Lazo GR, Chao S, Jellen EN, Carson ML et al (2013) SNP discovery and chromosome anchoring provide the first physically-anchored hexaploid oat map and reveal synteny with model species. PLoS ONE 8:E58068. 10.1371/journal.pone.0058068 10.1371/journal.pone.0058068 PMC3606164 23533580

[CR45] Othman RA, Moghadasian MH, Jones P (2011). Cholesterol-lowering effects of oat β-glucan. Nutr Rev.

[CR46] Roderick HW, Jones IT (1988). The effect of powdery mildew (*Erysiphe graminis* fsp *avenae*) on yield, yield components and grain quality of spring oats. Ann Appl Biol.

[CR47] Saintenac C, Cambon F, Aouini L, Verstappen E, Ghaffary SMT, Poucet T, Marande W, Berges H, Xu S, Jaouannet M (2021). A wheat cysteine-rich receptor-like kinase confers broad-spectrum resistance against *Septoria tritici* blotch. Nat Commun.

[CR56] Sánchez-Martín J, Keller B (2021) NLR immune receptors and diverse types of non-NLR proteins control race-specific resistance in *Triticeae*. Curr Opin Plant Biol 62:102053. 10.1016/j.pbi.2021.102053 10.1016/j.pbi.2021.102053 34052730

[CR54] Sánchez-Martín J, Rubiales D, Prats E (2011). Resistance to powdery mildew (*Blumeria graminis* f sp *avenae*) in oat seedlings and adult plants. Plant Pathol.

[CR55] Sánchez-Martín J, Widrig V, Herren G, Wicker T, Zbinden H, Gronnier SL, Praz CR, Heuberger M, Kolodziej MC, Isaksson J, Steuernagel B, Karafiátová M, Doležel J, Zipfel C, Keller B (2021). Wheat *Pm4* resistance to powdery mildew is controlled by alternative splice variants encoding chimeric proteins. Nat Plants.

[CR48] Sanz MJ, Jellen EN, Loarce Y, Irigoyen ML, Ferrer E, Fominaya A (2010). A new chromosome nomenclature system for oat (*Avena sativa* L and *A byzantine* C Koch) based on FISH analysis of monosomic lines. Theor Appl Genet.

[CR49] Schultink A, Steinbrenner AD (2021). A playbook for developing disease-resistant crops through immune receptor identification and transfer. Curr Opin Plant Biol.

[CR50] Singh RJ, Kolb FL (1991). Chromosomal interchanges in six hexaploid oat genotypes. Crop Sci.

[CR51] Sowa S, Paczos-Grzęda E (2020). Identification of molecular markers for the *Pc39* gene conferring resistance to crown rust in oat. Theor Appl Genet.

[CR52] Steele JFC, Hughes RK, Banfield MJ (2019). Structural and biochemical studies of an NB-ARC domain from a plant NLR immune receptor. PLoS ONE.

[CR53] Stein N, Herren G, Keller B (2001). A new DNA extraction method for high-throughput marker analysis in a large-genome species such as *Triticum aestivum*. Plant Breed.

[CR58] Tinker NA, Kilian A, Wight CP, Heller-Uszynska K, Wenzl P, Rines HW (2009). New DArT markers for oat provide enhanced map coverage and global germplasm characterization. BMC Genomics.

[CR57] Tinker NA, Bekele WA, Hattori J (2016). Haplotag software for haplotype-based genotyping-by-sequencing analysis. G3 Genes Genomes Genet.

[CR59] Van Ooijen JW (2006) Joinmap 4: software for the calculation of genetic linkage maps in experimental populations. Kyazma BV, Wageningen, Netherlands

[CR60] Voorrips RE (2002). MapChart: software for the graphical presentation of linkage maps and QTLs. J Hered.

[CR62] Waters A (2020) Assembly and annotations for the OT3098 oat genome; *Avena sativa*—OT3098 v1, PepsiCo. https://wheat.pw.usda.gov/GG3/graingenes_downloads/oat-ot3098-pepsico

[CR61] Wang J, Chen T, Han M, Qian L, Li J, Wu M (2020). Plant NLR immune receptor Tm-22 activation requires NB-ARC domain-mediated self-association of CC domain. PLoS Pathog.

[CR63] Wimmer V, Albrecht T, Auinger HJ, Schön CC (2012). Synbreed: a framework for the analysis of genomic prediction data using R. Bioinformatics.

[CR64] Yan H, Bekele WA, Wight CP, Peng Y, Langdon T, Latta RG, Fu Y-B, Diederichsen A, Howarth CJ, Jellen EN, Boyle B, Wei Y, Tinker NA (2016). High-density marker profiling confirms ancestral genomes of *Avena* species and identifies D-genome chromosomes of hexaploid oat. Theor Appl Genet.

[CR65] Yu J, Herrmann MH (2006). Inheritance and mapping of a powdery mildew resistance gene introgressed from *Avena macrostachya* in cultivated oat. Theor Appl Genet.

[CR66] Zhang Z, Ersoz E, Lai CQ, Todhunter RJ, Tiwari HK, Gore MA, Bradbury PJ, Yu J, Arnett DK, Ordovas JM, Buckler ES (2010). Mixed linear model approach adapted for genome-wide association studies. Nat Genet.

