# Supplementary material for: Genetic mapping of the powdery mildew resistance gene Pm7 on oat chromosome 5D
Source: Theor Appl Genet. 2023 Mar 13;136(3):53. doi: 10.1007/s00122-023-04288-z (PMC10011287; doi:10.1007/s00122-023-04288-z)
Supplement: Supplementary file 1 — Supplementary file1 (DOCX 54 KB) [file 122_2023_4288_MOESM1_ESM.docx]

**
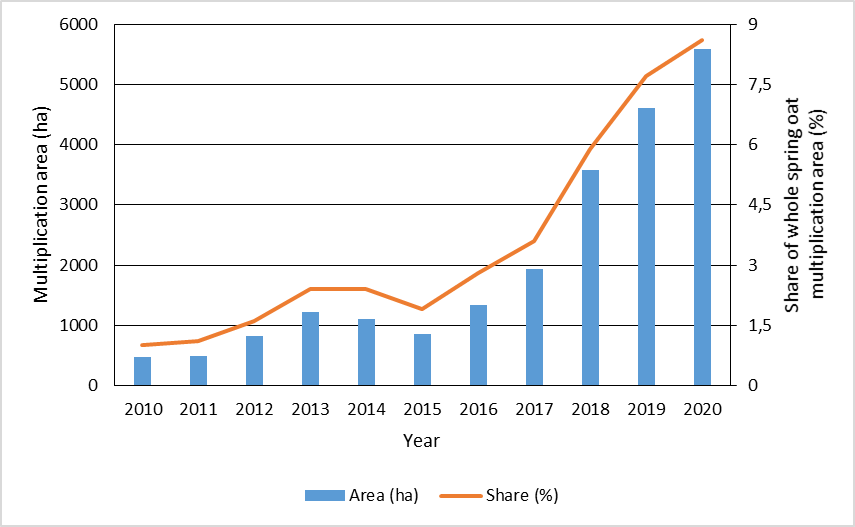
**

Figure S1: Multiplication area and share of *pm7*-oat varieties of the whole European spring oat multiplication (Source: Nordsaat Saatzucht GmbH)
