# Supplementary figures and images for: Genetic mapping of the powdery mildew resistance gene Pm7 on oat chromosome 5D
Source: Theor Appl Genet. 2023 Mar 13;136(3):53. doi: 10.1007/s00122-023-04288-z (PMC10011287; doi:10.1007/s00122-023-04288-z)

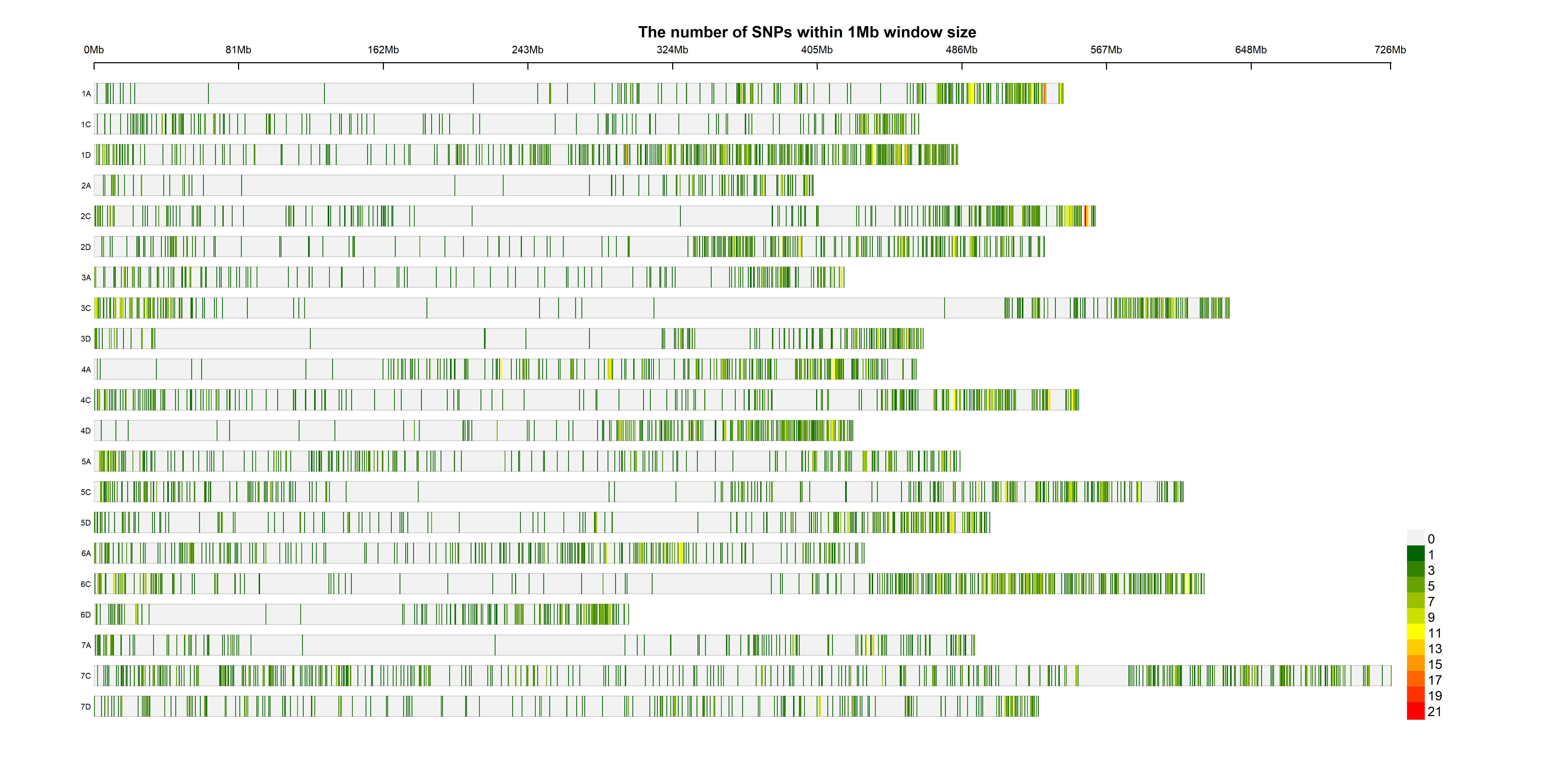

Supplement: Supplementary file 2 — Supplementary file2 (JPG 2697 KB) [file 122_2023_4288_MOESM2_ESM.jpg]

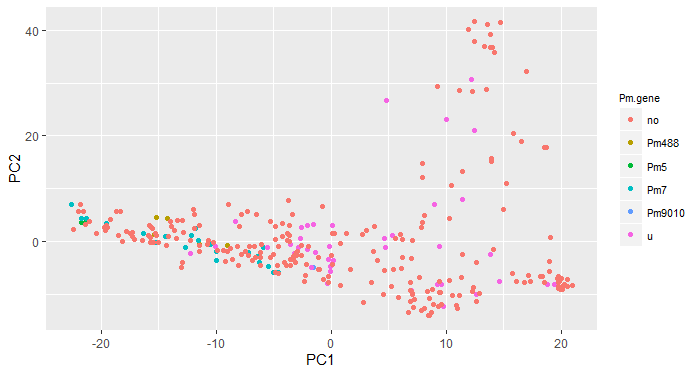

Supplement: Supplementary file 3 — Supplementary file3 (TIFF 769 KB) [file 122_2023_4288_MOESM3_ESM.tiff]

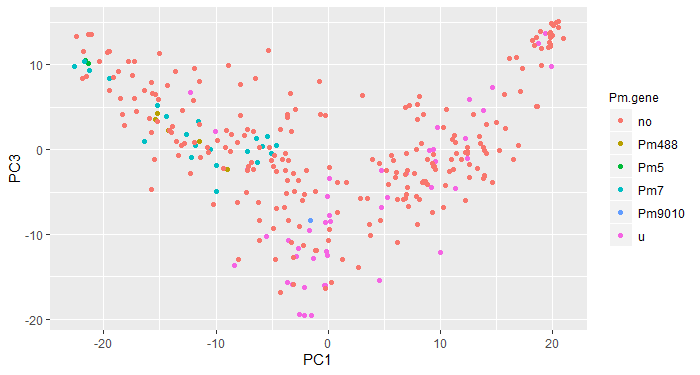

Supplement: Supplementary file 4 — Supplementary file4 (TIFF 769 KB) [file 122_2023_4288_MOESM4_ESM.tiff]

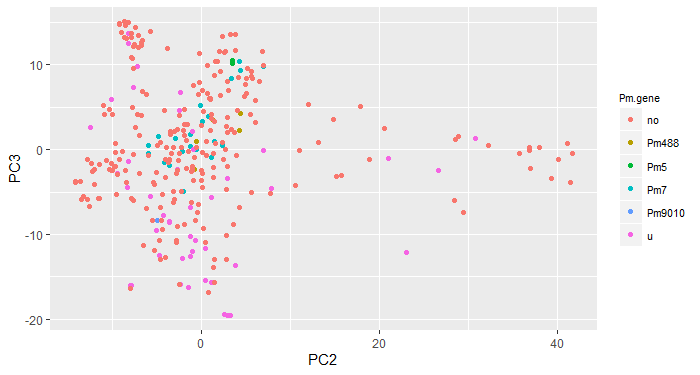

Supplement: Supplementary file 5 — Supplementary file5 (TIFF 769 KB) [file 122_2023_4288_MOESM5_ESM.tiff]

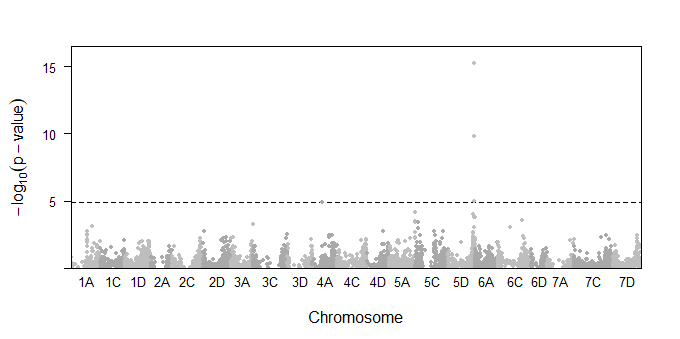

Supplement: Supplementary file 6 — Supplementary file6 (TIFF 676 KB) [file 122_2023_4288_MOESM6_ESM.tiff]

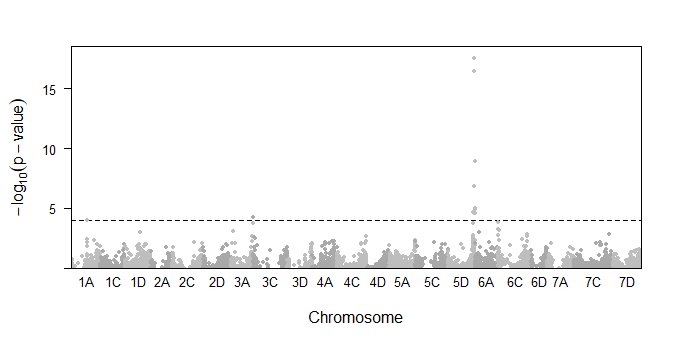

Supplement: Supplementary file 7 — Supplementary file7 (TIFF 676 KB) [file 122_2023_4288_MOESM7_ESM.tiff]
