# Supplementary material for: Genetic mapping of the powdery mildew resistance gene Pm7 on oat chromosome 5D
Source: Theor Appl Genet. 2023 Mar 13;136(3):53. doi: 10.1007/s00122-023-04288-z (PMC10011287; doi:10.1007/s00122-023-04288-z)
Supplement: Supplementary file 8 — Supplementary file8 (DOCX 14 KB) [file 122_2023_4288_MOESM8_ESM.docx]

**Table S1: Yield of oats in variety trials of the AHDB in the UK 2016-2020 (AHDB 2021)**

| **Trait** | | **Variety type** | |
| --- | --- | --- | --- |
|  |  | **pm7+ (n=4)** | **pm7- (n=5)** |
| Fungicide-treated yield (t/ha) | | 7,19 | 6,83 |
| Fungicide-treated yield (%) | | 100 | 100 |
| Untreated yield (t/ha) | | 6,78 | 5,92 |
| Untreated yield (%) | | 94 | 87 |

AHDB (2021) Recommended Lists for cereals and oilseeds (RL) harvest results (archive) (UK), https://ahdb.org.uk/knowledge-library/recommended-lists-for-cereals-and-oilseeds-rl-harvest-results-archive
